# From a single child to family planning: determinants of returning to ART to build the desired family in a monocentric Italian cohort

**DOI:** 10.1093/hropen/hoag063

**Published:** 2026-07-21

**Authors:** Francesco Viciglione, Valentina Immediata, Emanuela Morenghi, Carlo Alviggi, Annamaria Baggiani, Federico Cirillo, Paolo Emanuele Levi-Setti

**Affiliations:** Department of Biomedical Sciences, Humanitas University, Milan, Italy; Division of Gynecology and Reproductive Medicine, Department of Gynecology, Fertility Center, Istituto di Ricerca e Cura a Carattere Scientifico IRCCS Humanitas Research Hospital, Rozzano, Milan, Italy; Division of Gynecology and Reproductive Medicine, Department of Gynecology, Fertility Center, Istituto di Ricerca e Cura a Carattere Scientifico IRCCS Humanitas Research Hospital, Rozzano, Milan, Italy; Division of Gynecology and Reproductive Medicine, Department of Gynecology, Fertility Center, Istituto di Ricerca e Cura a Carattere Scientifico IRCCS Humanitas Research Hospital, Rozzano, Milan, Italy; Department of Public Health, University of Naples Federico II, Naples, Italy; Division of Gynecology and Reproductive Medicine, Department of Gynecology, Fertility Center, Istituto di Ricerca e Cura a Carattere Scientifico IRCCS Humanitas Research Hospital, Rozzano, Milan, Italy; Division of Gynecology and Reproductive Medicine, Department of Gynecology, Fertility Center, Istituto di Ricerca e Cura a Carattere Scientifico IRCCS Humanitas Research Hospital, Rozzano, Milan, Italy; Department of Biomedical Sciences, Humanitas University, Milan, Italy; Division of Gynecology and Reproductive Medicine, Department of Gynecology, Fertility Center, Istituto di Ricerca e Cura a Carattere Scientifico IRCCS Humanitas Research Hospital, Rozzano, Milan, Italy

**Keywords:** ART, family building, family planning, cryopreservation, live birth, reproductive decision-making, reproductive outcomes

## Abstract

**STUDY QUESTION:**

What factors are associated with couples’ returning to have ART after having a first ART-conceived child?

**SUMMARY ANSWER:**

Among returning couples, return was independently associated with younger maternal age, a singleton rather than twin first birth, and the availability of cryopreserved embryos and/or oocytes.

**WHAT IS KNOWN ALREADY:**

Long-term family-building trajectories after an initial ART birth remain poorly characterized, despite being essential for counseling couples aiming to achieve their intended family size in the context of declining fertility rates and delayed parenthood in high-income countries. Age-related biological constraints, limited fertility awareness, and overestimation of ART effectiveness contribute to unrealized reproductive intentions; nevertheless, the determinants of couples’ return to ART after a first live birth and their association with subsequent live births remain largely unexplored.

**STUDY DESIGN, SIZE, DURATION:**

Monocentric retrospective cohort study of 5852 couples achieving a first ART-conceived live birth (index birth) from cycles initiated between 2010 and 2020, with follow-up through December 2024 to evaluate return to ART for further family building and subsequent live birth outcomes. As data were retrieved from a complete institutional database, no loss to follow-up occurred.

**PARTICIPANTS/MATERIALS, SETTING, METHODS:**

Couples achieving a first ART-conceived live birth between 2010 and 2020 at a single fertility center in Italy were included. Donor cycles and PGT were excluded. Participants were followed until December 2024. Couples were classified as returners or non-returners and stratified according to cryopreserved oocyte and/or embryo storage status. Outcomes of interest were return to ART for further family building and live birth achievement among returning couples.

**MAIN RESULTS AND THE ROLE OF CHANCE:**

Overall, 1978 couples (33.8%) returned for further ART cycles; among returners, a second live birth was achieved by 49.3%. Return was significantly higher among couples with cryopreserved reproductive material compared with those without stored embryos or oocytes (41.0% vs 23.3%, *P* < 0.001), particularly among couples with cryopreserved embryos only or with both embryos and oocytes stored (41.6% and 44.3%, respectively). At multivariable analysis, independent predictors of return included lower maternal age (aOR 0.93 per year increase), male-factor infertility (aOR 1.26), singleton rather than twin first live birth (aOR 0.12 for twins), presence of cryopreserved material (aOR 2.21), and higher gestational age at delivery (aOR 1.03). Among couples who returned, the proportion achieving a second live birth varied markedly by cryopreservation status, ranging from 39.5% in couples without stored material to 64.6% in those with both embryos and oocytes available (*P* < 0.001). Few couples returned after a second live birth; however, the live birth proportion for a third child exceeded 50% when both embryos and oocytes were present.

**LIMITATIONS, REASONS FOR CAUTION:**

Psychosocial and economic determinants potentially influencing non-returners were not assessed, data on spontaneous pregnancies were unavailable, and cross-center migration could not be excluded. In addition, this monocentric study was conducted in Italy, where regulatory and cultural aspects of ART may influence treatment access and reproductive decision-making, potentially limiting generalizability.

**WIDER IMPLICATIONS OF THE FINDINGS:**

ART counseling may benefit from being tailored to couples’ long-term reproductive goals. Treatment strategies aimed at increasing the number of embryos and/or oocytes cryopreserved at younger ages can facilitate their use at more advanced reproductive ages. This may help couples achieve their desired family size.

**FUNDING:**

This research received no specific grant from any funding agency in the public, commercial, or not-for-profit sectors, and was supported by institutional resources from the Division of Gynecology and Reproductive Medicine, Department of Gynecology, Fertility Center, IRCCS Humanitas Research Hospital, Rozzano. Milan, Italy.

**DISCLOSURES:**

The authors declare no competing interests.

**TRIAL REGISTRATION NUMBER:**

NCT07458165

WHAT DOES THIS MEAN FOR PATIENTS? This study looks at whether couples who have a baby through fertility treatment go on to come back for further care to have another child, and what makes this more or less likely.

Many people who use fertility treatment hope to have more than one child, but little is known about who comes back after their first baby and who is more likely to succeed.

In this study, researchers looked at over 5800 couples who had a first baby through fertility treatment at one center in Italy. About one in three couples came back for more treatment. This was more likely when the mother was younger, when the first baby was a single birth rather than twins, and when the couple still had frozen eggs or embryos stored from before. Having frozen eggs or embryos also made it more likely that a second baby would be born. This suggests that storing eggs or embryos may help couples who hope to grow their family later on.

## Introduction

Over the last three decades, a consistent decline in fertility levels has been observed in most high-income countries (Billari and Kohler, 2004; Fauser *et al.*, 2024). In this demographic context, increasing attention has been devoted to reproductive intentions, with debate focusing on whether declining fertility reflects delayed parenthood or a genuine reduction in individuals’ desired family size (Kocourková and Šťastná, 2021; Lazzari *et al.*, 2025).

The concept of the fertility gap describes the discrepancy between the planned and the actual number of children and is closely related to the non-attainment of intended reproductive goals (Slabá *et al.*, 2024). In Italy, data from the National Institute of Statistics (ISTAT) show that, among individuals intending to have children, 56.1% report wanting at least two, in contrast with a total fertility rate that continues to decline, reaching 1.18 children per woman in 2024 (Istituto Nazionale di Statistica (ISTAT), 2025). This divergence is largely attributed to postponement of childbearing, particularly among women, driven by cultural, social, and economic factors (Schmidt *et al.*, 2012).

Age-related analyses from US population data demonstrate that the mismatch between fertility desires and intentions increases progressively with advancing age (Badolato, 2026). Among individuals who desire additional children and are physically able to have them, this gap rises from ∼6% in the 25–29 years age group to 15% among those aged 35–39 years and over 30% among those aged 40–44 years, with women consistently showing a wider gap than men across all age groups (Badolato, 2026).

Several determinants contribute to delayed childbearing. Using data from the Czech registry, Slabá *et al.* (2024) identified the absence of a suitable partner, infertility-related difficulties, financial constraints, and health problems as the main drivers, with partner-related factors exerting the strongest effect. Limited fertility awareness may further exacerbate postponement (Pedro *et al.*, 2018; Ren *et al.*, 2023). A systematic review by Pedro *et al.* (2018), including 71 studies from 26 countries, showed that fertility knowledge in the general population remains suboptimal. Population-based studies further report frequent misconceptions regarding the definition of infertility and poor awareness of the timing of the fertile window of the menstrual cycle (Bunting and Boivin, 2008; Bunting *et al.*, 2013). Furthermore, many individuals underestimate the magnitude of age-related fertility decline while overestimating the effectiveness of ARTs (Lampic *et al.*, 2006).

The World Health Organization (WHO) estimates that one in six individuals of reproductive age experiences fertility problems and recognizes the achievement of one’s desired family size as a fundamental reproductive right that may be compromised by infertility (Zegers-Hochschild *et al.*, 2013; Mburu *et al.*, 2026).

ART represents a key therapeutic option for infertile couples, with IVF being the most widely used approach (Zegers-Hochschild *et al.*, 2013; Istituto Nazionale di Statistica (ISTAT), 2025). Ovarian stimulation (OS) and transvaginal oocyte retrieval (TVOR) are essential for embryo generation using autologous gametes but may be associated with ovarian hyperstimulation syndrome (OHSS), bleeding, infection, and, rarely, severe adverse events including hemoperitoneum, organ injury, vascular, and anesthetic complications (Levi-Setti *et al.*, 2018). When clinically feasible, obtaining multiple embryos from a single stimulation cycle may reduce the need for repeated treatments and additional OS and TVOR; however, Italian Ministry of Health guidelines recommend the cryopreservation of only the number of embryos strictly necessary (Ministero della Salute Italiano, 2024). Within this clinical and regulatory framework, evidence on return for further treatment after a first ART live birth and subsequent family-building outcomes remains limited. A recent systematic review reported return rates to ART for a second child ranging from 25% to 50% across different contexts, with younger age and availability of cryopreserved embryos consistently associated with higher return, underscoring the need for further large cohort studies to better characterize determinants and outcomes (Li Piani *et al.*, 2026). The study aimed to define the profile of couples who returned for further ART treatment after a first live birth, identify factors associated with return, and assess the proportion of couples achieving a subsequent live birth among returning couples.

## Materials and methods

### Ethical approval

The study was approved by the Institutional Ethics Committee (protocol number 4773). All patients undergoing ART treatment provided written informed consent for the use of anonymized clinical data for research purposes. The study was registered at ClinicalTrials.gov (ID: NCT07458165) prior to data extraction and analysis.

### Study design and setting

This single-center retrospective cohort study was conducted at the Humanitas Fertility Center, a tertiary university-affiliated ART center in Rozzano, Milan, Italy. All couples who achieved a first live birth following ART between January 2010 and December 2020 were identified and followed until December 2024. As index births occurred over an 11-year recruitment window, this resulted in a variable follow-up duration of approximately 4–15 years per couple, applied identically to returners and non-returners irrespective of maternal age reached by the end of follow-up.

### Study population

Eligible participants were heterosexual couples in which the female partner was aged 18–46 years. This upper age limit reflects the Italian regulatory framework during the study period. Treatment cycles were performed both within the public healthcare system and on a private basis. Couples undergoing treatment cycles involving preimplantation genetic testing for aneuploidy (PGT-A) or the use of donor gametes were excluded. After application of the eligibility criteria, the final study cohort comprised 5852 couples. The index birth was defined as the first live birth achieved following ART treatment performed at our center. During follow-up, couples were categorized as returners, defined as those who initiated at least one additional treatment cycle (including OS, frozen embryo transfer (FET), or frozen oocyte thaw cycles) following the index birth, or non-returners, defined as those who did not pursue additional treatment. Couples attending clinical consultations without proceeding to treatment were included in the latter category. Among returners, follow-up for a subsequent live birth was open-ended up to the same study end date, with no maximum time window imposed.

### Outcomes

The outcomes of interest were the characterization of couples who returned for further ART treatment after the first live birth and the identification of factors associated with return, and the proportion of couples achieving a subsequent live birth among those who returned.

### Data collection and variables

Clinical, demographic, and treatment-related variables were extracted from the internal database, including maternal age at index birth, BMI, infertility diagnosis, number of OS cycles before the index birth, obstetric outcomes, gestational age at delivery, maternal and fetal complications, and twin birth. Analyses were prespecified to be stratified by maternal age at the index birth and according to the availability of cryopreserved reproductive material, the latter classified into four groups: no cryopreserved embryos or oocytes (NOE), oocytes only (OO), embryos only (OE), or both oocytes and embryos (BOE).

### Statistical analysis

Continuous variables are presented as mean ± SD or median (interquartile range), as appropriate, and categorical variables as number and percentage. Baseline characteristics were compared between returners and non-returners. Univariable logistic regression analysis was performed to identify variables associated with return for further treatment. Variables with *P*-value <0.10 at univariable analysis and those considered clinically relevant were included in multivariable logistic regression models to estimate adjusted odds ratios (aORs) and 95% CIs. Among returners, live birth proportion was calculated to evaluate subsequent reproductive outcomes and analyses were stratified according to maternal age at the index birth and availability of cryopreserved reproductive material. All statistical analyses were performed using Stata version 18.0 (Stata Corp. 2023, College Station, TX, USA). All tests were two-sided and *P *< 0.05 was considered statistically significant.

## Results

A total of 5852 couples who achieved a first live birth after ART were followed until December 2024. Among them, 1978 (33.8%) returned for further treatment, whereas 3874 (66.2%) did not. Baseline characteristics are reported in Table 1.

**Table 1.** Baseline characteristics of couples according to return for further ART treatment.

	Returners (n = 1978)	Non-returners (n = 3874)	OR (95% CI)	*P*-value
**Female age at birth index (years), mean ± SD**	34.5 ± 3.7	35.6 ± 3.9	0.93 (0.91–0.94)	<0.001
**BMI (kg/m²), mean±SD**	22.0 ± 3.1	22.1 ± 3.3	1.00 (0.98–1.01)	0.804
**Male factor, n (%)**	903 (45.65%)	1465 (37.82%)	1.38 (1.24–1.54)	<0.001
**No. of OS before first birth, mean±SD**	1.52 ± 0.89	1.49 ± 0.83	1.04 (0.98–1.11)	0.238
**First twin birth, n (%)**	54 (2.73%)	701 (18.10%)	0.13 (0.10–0.17)	<0.001
**Remaining frozen reproductive material at index birth, n (%)**	1424 (71.99%)	2049 (52.89%)	2.29 (2.04–2.57)	<0.001
**NOE, n (%)**	554 (28.01%)	1825 (47.11%)	Ref	
**OO, n (%)**	82 (4.15%)	204 (5.27%)	1.32 (1.01–1.74)	0.044
**OE, n (%)**	1065 (53.84%)	1497 (38.64%)	2.34 (2.07–2.65)	<0.001
**BOE, n (%)**	277 (14.00%)	348 (8.98%)	2.62 (2.18–3.15)	<0.001
**Maternal–fetal complications at index birth, n (%)**	467 (23.61%)	756 (19.51%)	1.27 (1.12–1.45)	<0.001
Maternal, n (%)	420 (21.23%)	672 (17.35%)	1.28 (1.12–1.47)	<0.001
Fetal, n (%)	100 (5.06%)	193 (4.98%)	1.02 (0.79–1.30)	0.903
**Gestational age at delivery (weeks), mean ± SD^a^**	38.8 ± 2.3	38.1 ± 2.6	1.14 (1.11–1.17)	<0.001
<28 weeks, n (%)	22 (1.16%)	34 (0.92%)	1.24 (0.72–2.14)	0.430
28 to <32 weeks, n (%)	14 (0.74%)	106 (2.87%)	0.25 (0.14–0.44)	<0.001
≥32 weeks, n (%)	1853 (98.09%)	3558 (96.21%)	Ref	

Data are presented as mean±SD or n (%). Odds ratios (OR) and 95% CIs were obtained from univariable logistic regression. *P*-values were calculated as described in the Materials and Methods section. Index birth refers to the first live birth following ART.

a Gestational age at delivery was available for 1889 returners and 3698 non-returners.

BOE, both oocytes and embryos in storage; n, number; NOE, neither oocytes nor embryos in storage; OE, embryos only in storage; OO, oocytes only in storage; OR, odds ratio; OS, ovarian stimulation.

Returners were younger at the index birth (mean 34.5 ± 3.7 vs 35.6 ± 3.9 years; *P *< 0.001) and more frequently had cryopreserved reproductive material remaining (71.9% vs 52.9%; *P *< 0.001). Twin delivery was markedly less common among returners (2.7% vs 18.1%; *P *< 0.001). Gestational age at delivery was slightly higher in returners (mean 38.8 ± 2.3 vs 38.1 ± 2.6 weeks; *P *< 0.001). Male-factor infertility (45.6% vs 37.8%; *P *< 0.001) and maternal–fetal complications (23.6% vs 19.5%; *P *< 0.001) differed between groups, although differences were modest. No differences were observed for BMI or number of OS cycles. Privately funded cycles accounted for 4.6% of the cohort and were similarly distributed between returners and non-returners (4.89% vs 4.18%; *P* = 0.207).

**Figure 1.**
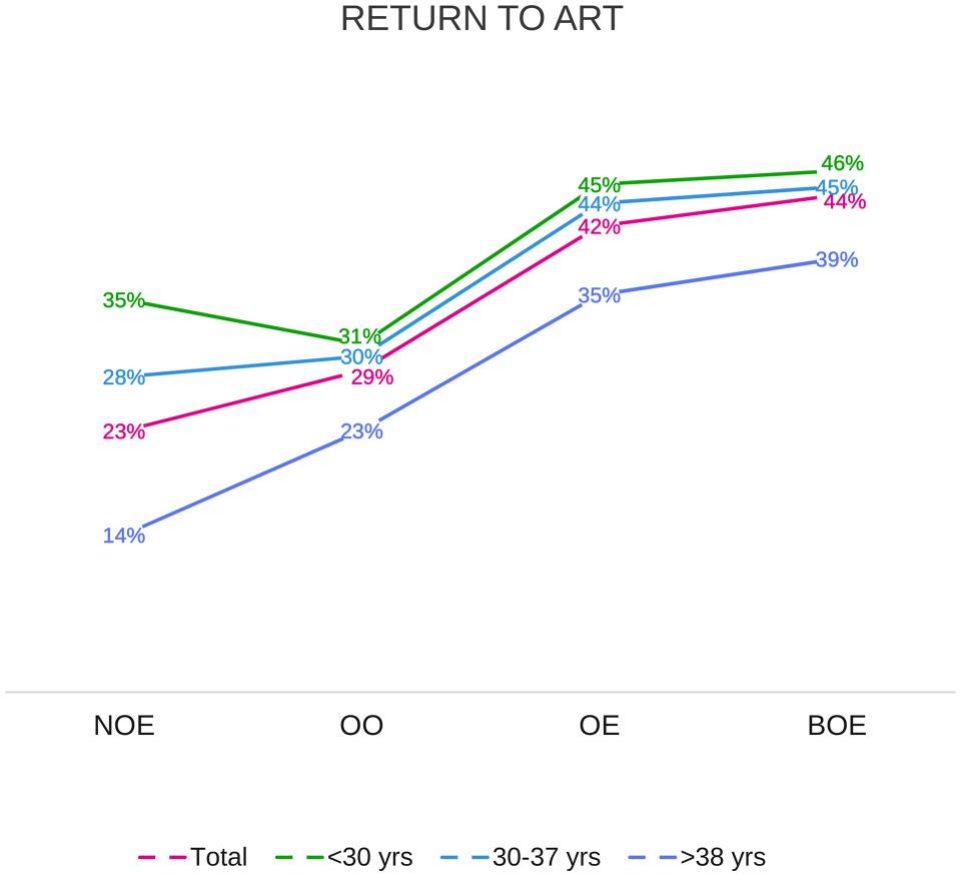
**Return for further ART treatment for a second child, total and stratified by age group**. Return proportion stratified by age group (<30, 30–37, ≥38 years) and availability of cryopreserved reproductive material. Percentages refer to the proportion of couples returning for further ART treatment. NOE, neither oocytes nor embryos in storage; OO, oocytes only in storage; OE, embryos only in storage; BOE, both oocytes and embryos in storage.

The proportion of returning couples varied according to cryopreservation status (Fig. 1): 23.3% in NOE, 28.7% in OO, 41.6% in OE, and 44.3% in BOE (*P *< 0.001). An age-related decline was observed across all categories. Among women <30 years, the proportion of returners ranged from 35.0% (NOE) to 46.5% (BOE); in women aged 30–37 years from 28.1% to 45.1%; and in women ≥38 years from 14.0% to 38.6%. At comparable ages, the presence of cryopreserved material increased the probability of further treatment, whereas in NOE the proportion of returning couples remained relatively low irrespective of age (Fig. 1).

**Figure 2.**
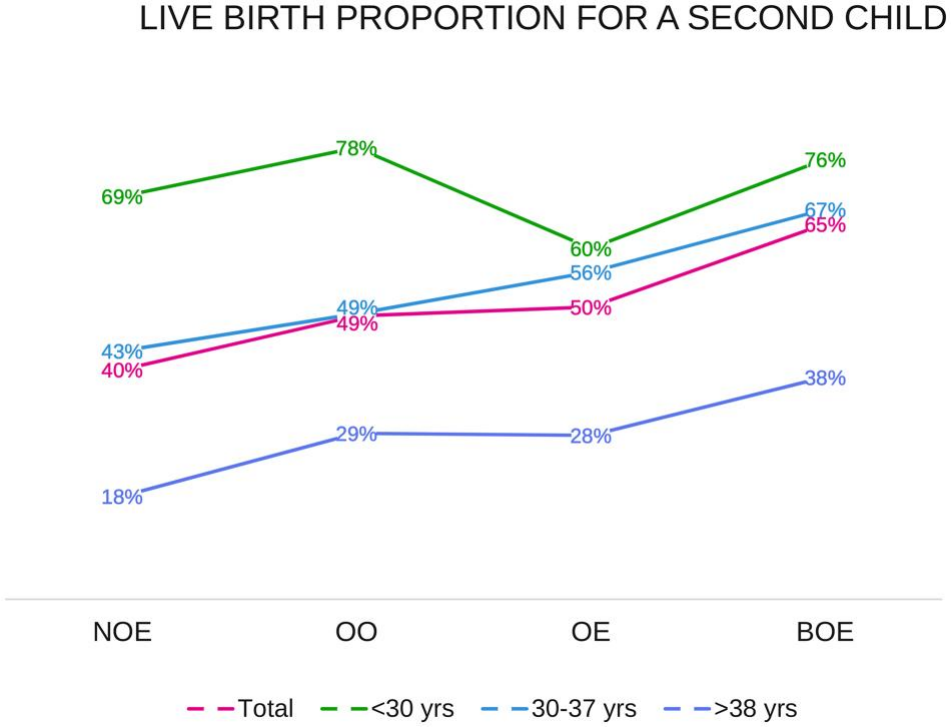
**Total and age-stratified live birth proportion for a second live birth among returning couples**. Live birth proportion represents the probability of achieving a subsequent live birth after the index live birth among couples who returned for further ART treatment. Analyses are stratified by age group (<30, 30–37, ≥38 years) and availability of cryopreserved reproductive material. NOE, neither oocytes nor embryos in storage; OO, oocytes only in storage; OE, embryos only in storage; BOE, both oocytes and embryos in storage.

Among returners, the proportion achieving a second live birth differed by cryopreservation status (Fig. 2), being 39.5% in NOE, 48.8% in OO, 50.3% in OE, and 64.6% in BOE (*P *< 0.001). Age-stratified analyses showed a progressive reduction with advancing age: in women <30 years live birth proportion ranged from 69.4% to 75.8%, in women 30–37 years from 42.8% to 67.1%, and in women ≥38 years from 17.7% to 38.2% (Fig. 2). Baseline and treatment characteristics of couples according to cryopreservation status are reported in Table 2. Among couples who achieved a second live birth, the median time elapsed between the first and second live birth was 36 months (range 9–113), with no significant difference according to cryopreservation status (*P* = 0.620).

**Table 2.** Baseline and treatment characteristics according to availability of cryopreserved reproductive material after the index live birth.

	Overall (n = 5852)	No remaining frozen material (n = 2379)	Remaining cryopreserved oocytes/embryos (n = 3473)	*P*-value
**Female age at OS (years), mean±SD**	35.2 ± 3.8	35.9 ± 3.8	34.5 ± 3.8	<0.001
**BMI (kg/m²), mean±SD**	22.0 ± 3.2	22.2 ± 3.4	21.9 ± 3.1	0.022
**Male-factor infertility, n (%)**	2368 (40.46%)	840 (35.31%)	1528 (44.00%)	<0.001
**Previous OS before index birth, median (range), mean±SD**	1.50 ± 0.85	1 (1-7); 1.64 ± 0.95	1 (1-8); 1.40 ± 0.76	<0.001
**Oocytes retrieved, mean±SD**	11.7 ± 6.1	8.58 ± 4.56	13.78 ± 6.16	<0.001
**Twin birth at index delivery, n (%)**	755 (12.90%)	295 (12.41%)	460 (13.25%)	0.347
**Art procedure at index birth, n (%)**				<0.001
Fresh ET	3545 (60.58%)	1626 (68.35%)	1919 (55.25%)	
ET following oocyte thawing	100 (1.71%)	59 (2.48%)	41 (1.18%)	
FET	2207 (37.71%)	694 (29.17%)	1513 (43.56%)	
**Additional ART cycles after first birth, n (%)**	1978 (33.80%)	554 (23.30%)	1424 (41.00%)	<0.001
**Second live birth, n (%)**	974 (16.64%)	219 (9.21%)	755 (21.74%)	<0.001

Data are presented as mean±SD, median (range), or n (%). For selected variables, both median (range) and mean±SD are reported to better describe distribution. *P*-values refer to comparisons between groups as described in the Materials and Methods section. Index birth refers to the first live birth achieved through ART.

ET, embryo transfer; FET, frozen embryo transfer; OS, ovarian stimulation.

At multivariable logistic regression (Table 3), return was independently associated with maternal age (aOR 0.93 per year; 95% CI 0.92–0.95; *P *< 0.001), twin index birth (aOR 0.12 vs singleton; 95% CI 0.09–0.16; *P *< 0.001), and availability of cryopreserved reproductive material (aOR 2.21; 95% CI 1.95–2.50; *P *< 0.001). Male-factor infertility (aOR 1.26; 95% CI 1.12–1.42; *P *< 0.001), maternal–fetal complications (aOR 1.29; 95% CI 1.12–1.49; *P *< 0.001), and higher gestational age at delivery (aOR 1.03 per week; 95% CI 1.01–1.06; *P *= 0.021) were also independently associated with return for further treatment.

**Table 3.** Multivariable logistic regression analysis of factors associated with return for further ART treatment.

	aOR (95% CI)	*P*-value
**Female age at birth index**	0.93 (0.92–0.95)	<0.001
**Male factor**	1.26 (1.12–1.42)	<0.001
**First twin birth**	0.12 (0.09–0.16)	<0.001
**Remaining frozen biological material at birth index**	2.21 (1.95–2.50)	<0.001
**Maternal–fetal complications at index birth**	1.29 (1.12–1.49)	<0.001
**Gestational age at delivery**	1.03 (1.01–1.06)	0.021

Adjusted odds ratios (aOR) and 95% CIs were obtained from multivariable logistic regression analysis. Index birth refers to the first live birth following ART.

aOR, adjusted odds ratio.

Only a limited proportion of couples returned after the second live birth. Nevertheless, findings for a third child mirrored those observed for the second live birth, with higher rates in the presence of cryopreserved material and at younger maternal age. Detailed results are provided in Supplementary Tables S1 and S2.

## Discussion

This study examined whether couples return for further attempts to have another child after a first ART-conceived live birth and the factors influencing this decision. Among those who returned, we evaluated the cumulative probability of achieving a subsequent live birth. Overall, approximately one-third of couples returned after a first ART-conceived live birth.

The availability of cryopreserved reproductive material emerged as the most consistent determinant of return, with progressively higher proportions observed from couples without stored material to those with both oocytes and embryos available, a pattern that remained evident across maternal age groups. Maternal age was also strongly associated with return, with younger couples more likely to pursue further attempts, while a twin index birth markedly reduced the likelihood of returning. These findings are consistent with those reported in the systematic review by Li Piani *et al.* (2026), which identified younger age and availability of cryopreserved embryos as key determinants of return across different contexts.

Among returning couples, the cumulative probability of achieving a second live birth was substantial and closely associated with both maternal age and cryopreservation status, reaching its highest levels when reproductive material preserved at younger ages was available. Comparable trends were observed for attempts toward a third child, although only a minority of couples pursued further treatment.

The observed association reflects the biological consequences of reproductive aging. Increasing maternal age is associated with higher aneuploidy rates and reduced oocyte competence, therefore requiring a larger oocyte cohort to obtain an adequate number of euploid blastocysts (Vaiarelli *et al.*, 2018; Namath *et al.*, 2025). In this context, the availability of gametes or embryos created at younger ages represents a preserved reproductive potential rather than a simple reserve of treatment material. This biological advantage explains why the benefit of cryopreserved material persisted even at older maternal ages, as reproductive competence is determined at generation rather than at the time of use. Importantly, the association was only minimally affected after adjustment for clinical characteristics, indicating that the availability of cryopreserved material represents an independent determinant of couples’ return rather than a simple marker of favorable prognosis.

Beyond biological prognosis, several findings suggest that the decision to pursue another child is also influenced by prior reproductive experience. Couples who had a twin index birth were markedly less likely to return, likely reflecting early fulfilment of desired family size. Additionally, twin parenthood has been associated with a modestly increased risk of relationship dissolution, which may further contribute to the lower return observed in this group (Jena *et al.*, 2011).

The association with male-factor infertility may also reflect relatively preserved female reproductive potential in these couples, although paternal age-related changes in sperm competence could contribute (Elkhatib *et al.*, 2025). Obstetric experience was likewise relevant, as gestational age at delivery and prior complications were associated with the decision to attempt another pregnancy. Together, these observations support the concept that return to treatment reflects not only reproductive potential but also couples’ perception of feasibility and family planning intentions. Indeed, population-level data indicate that even among mothers who successfully conceive through ART, completed family size remains smaller than that achieved after natural conception, with a permanent fertility gap persisting through the end of the reproductive lifespan (Choi *et al.*, 2023).

These findings should also be interpreted in the context of the evolution of contemporary ART practice. Improved vitrification and warming techniques and refined embryo assessment increase IVF efficiency, maximizing outcomes from a single OS cycle (Anderson *et al.*, 2020; Practice Committees of the American Society for Reproductive Medicine and the Society for Reproductive Biologists and Technologists, 2021; Walker *et al.*, 2024; Coticchio *et al.*, 2025). Advances in clinical practice have also improved the safety of OS, making the management of high-response patients more controlled and predictable (Feferkorn *et al.*, 2023; Ata *et al.*, 2026). These advances have enabled the storage of larger amounts of viable reproductive material.

Furthermore, obstetric and perinatal outcomes after ART have become increasingly favorable and may further support the pursuit of additional pregnancies (Karavani *et al.*, 2022; Pinborg *et al.*, 2023). In this context, a previous twin pregnancy, although it may allow earlier achievement of reproductive goals, remains a high-risk condition for both mother and offspring, reinforcing the recommendation to favor single-embryo transfer to minimize avoidable maternal and perinatal complications (Khalil *et al.*, 2016; Alteri *et al.*, 2024).

Despite the availability of cryopreserved material, a substantial proportion of couples did not return for further treatment, indicating that reproductive decision-making cannot be explained by biological potential alone. The reasons underlying non-return remain unclear, as psychosocial and economic factors, changes in relationship status, possible spontaneous pregnancies, and treatment cycles initiated at other fertility centers following the index birth were not captured. Notably, inter-center transfer of cryopreserved oocytes and embryos is permitted in Italy, although the associated administrative burden may favor return to the originating center, particularly among couples with stored material. Studies on treatment discontinuation suggest that non-return is multifactorial, encompassing poor prognosis, spontaneous conception, transfer to other centers, and psychological distress (Brandes *et al.*, 2009; Scognamiglio *et al.*, 2025). In couples who have already achieved a live birth, fulfilment of reproductive goals, spontaneous conception, and the desire to avoid further OS may represent more prominent drivers of non-return than poor prognosis or treatment discontinuation for logistical reasons, though this remains to be formally investigated.

Furthermore, socioeconomic factors have been shown to independently influence treatment continuation after ART, with women of lower socioeconomic position less likely to persist after unsuccessful attempts (Uggerhøj *et al.*, 2026). Moreover, socioeconomic disadvantage reduces the likelihood of achieving a desired family size, with ART mothers from lower socioeconomic backgrounds reaching a smaller completed family size compared to their more advantaged counterparts (Choi *et al.*, 2023). The potential influence of clinical counseling on the decision to return could not be assessed, as consultation visits not followed by treatment were not systematically recorded. Finally, the monocentric design and the Italian regulatory and cultural context may further limit generalizability to other healthcare systems.

The strengths of this study include the large cohort size and the extended follow-up, enabling evaluation of long-term reproductive trajectories after an ART-conceived live birth in a real-world clinical setting. The findings have practical implications for treatment planning.

Within the Italian regulatory framework, only the number of embryos strictly necessary for treatment may be generated (Ministero della Salute Italiano, 2024). In clinical practice, this number is estimated on the basis of couples’ reproductive prognosis and maternal age, with the aim of maximizing the cumulative probability of achieving at least one live birth while remaining within regulatory constraints. In the absence of PGT-A, embryo competence cannot be directly assessed, making this estimation inherently uncertain; notably, even when euploid blastocysts are confirmed, multiple transfers may be required to achieve optimal cumulative live-birth rates (Gill *et al.*, 2024). In particular, the probability of obtaining a euploid blastocyst decreases with advancing maternal age, often requiring a larger oocyte cohort and individualized treatment planning (Vaiarelli *et al.*, 2018; Namath *et al.*, 2025). In this setting, tools such as the ART Calculator, which estimate the number of mature oocytes required to obtain a euploid blastocyst based on couple-specific characteristics, may help guide treatment strategy (Esteves *et al.*, 2019).

These findings support counseling oriented toward long-term family building rather than a single live birth, integrating prognosis with couples’ reproductive intentions. Treatment planning should also consider the creation of surplus embryos and potential changes in couple status, including separation, which does not appear to be increased among couples undergoing ART (Martins *et al.*, 2018). Further research is needed to clarify how biological and social factors interact in shaping long-term reproductive choices.

## Supplementary Material

hoag063_Supplementary_Data

## Data Availability

The data underlying this article cannot be shared publicly for reasons of patient privacy and the terms of the Institutional Ethics Committee approval (protocol number 4773). Data may be shared with qualified researchers upon reasonable request to the corresponding author.
