# Supplementary material for: From a single child to family planning: determinants of returning to ART to build the desired family in a monocentric Italian cohort
Source: Hum Reprod Open. 2026 Jul 21;2026(3):hoag063. doi: 10.1093/hropen/hoag063 (PMC13476075; doi:10.1093/hropen/hoag063)
Supplement: Supplementary file 1 [file hoag063_supplementary_data.docx]

**Supplementary Table S1. Return for further ART treatment for a third child after the second live birth according to maternal age and cryopreservation status**

|  | Total | <30 years | 30–37 years | ≥ 38 years |
| --- | --- | --- | --- | --- |
| NOE | 11/219 (5.02%) | 1/7 (14.29%) | 6/120 (5.00%) | 4/92 (4.35%) |
| OO^a^ | 4/40 (10.00%) | 0/0 | 3/26 (11.54%) | 1/14 (7.14%) |
| OE | 23/536 (4.29%) | 3/18 (16.67%) | 12/270 (4.44%) | 8/248 (3.23%) |
| BOE | 14/179 (7.82%) | 1/8 (12.50%) | 11/104 (10.58%) | 2/67 (2.99%) |

Values are expressed as number of returning couples/total number of couples with a second live birth (%). Age refers to maternal age at the index birth. Index birth indicates the first live birth achieved through ART. ^a^Percentages were not calculated when no eligible couples were present in a subgroup.

ART, assisted reproductive technology; BOE, both oocytes and embryos in storage; NOE, neither oocytes nor embryos in storage; OE, embryos only in storage; OO, oocytes only in storage.

**Supplementary Table S2. Live birth proportion for a third child among couples returning after the second live birth according to maternal age and cryopreservation status**

|  | Total | <30 years | 30–37 years | ≥ 38 years |
| --- | --- | --- | --- | --- |
| NOE | 6/11 (54.55%) | 1/1 (100%) | 5/6 (83.33%) | 0/4 (0%) |
| OO^a^ | 1/4 (25.00%) | 0/0 | 1/3 (33.33%) | 0/1 (0%) |
| OE | 9/23 (39.13%) | 2/3 (66.67%) | 6/12 (50.00%) | 1/8 (12.50%) |
| BOE | 8/14 (57.14%) | 1/1 (100%) | 6/11 (54.55%) | 1/2 (50.00%) |

Values are expressed as number of third live births/number of couples returning for further ART treatment after the second live birth (%). Age refers to maternal age at the index birth. Index birth indicates the first live birth achieved through ART. ^a^Percentages were not calculated when no couples returned for treatment.

ART, assisted reproductive technology; BOE, both oocytes and embryos in storage; NOE, neither oocytes nor embryos in storage; OE, embryos only in storage; OO, oocytes only in storage.
